# Supplementary material for: Accuracy of consensual stereotypes in moral foundations: A gender analysis
Source: PLoS One. 2020 Mar 5;15(3):e0229926. doi: 10.1371/journal.pone.0229926 (PMC7058411; doi:10.1371/journal.pone.0229926)
Supplement: S1 Appendix — (DOCX) [file pone.0229926.s001.docx]

**Appendix A**


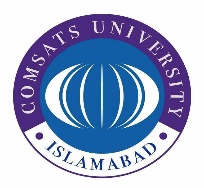


Informed Consent Form

Dear Participant,

Thank you so much for your willingness to participate in my research titled “**Accuracy of Consensual Stereotypes in Moral Foundations: A Gender Analysis**”. The purpose of my research is to investigate differences in moral psychology of men and women and to assess the accuracy of the stereotypes about morality that they have of each other.

I confirm that (please tick box as appropriate):

| 1. | I have read and understood the information about the project/research. | 🞏 |
| --- | --- | --- |
| 2. | I have been given the opportunity to ask questions about my participation. | 🞏 |
| 3. | I voluntarily agree to participate in the project. | 🞏 |
| 4. | I understand I can withdraw at any time without giving reasons and that I will not be penalised for withdrawing nor will I be questioned on why I have withdrawn. | 🞏 |
| 5. | The procedures regarding confidentiality have been clearly explained | 🞏 |
| 6. | I have been given information about the protocols for data collection. | 🞏 |
| 7. | The use of the data in research, publications, sharing and archiving has been explained. | 🞏 |
| 8. | I understand that other researchers will have access to this data only if they agree to preserve the confidentiality of the data and if they agree to the terms I have specified in this form. | 🞏 |
| 9. | I, along with the Researcher, agree to sign and date this informed consent form. | 🞏 |

**Participant:**

________________________ ___________________________ ________________

Name of Participant Signature Date

**Researcher:**

________________________ ___________________________ ________________

Name of Researcher Signature Date
