# Supplementary material for: Accuracy of consensual stereotypes in moral foundations: A gender analysis
Source: PLoS One. 2020 Mar 5;15(3):e0229926. doi: 10.1371/journal.pone.0229926 (PMC7058411; doi:10.1371/journal.pone.0229926)
Supplement: Attachment — Submitted filename: Response to reviewers.docx [file pone.0229926.s003.docx]

**Rebuttal Letter**

**Comments**

1. Is the manuscript technically sound, and do the data support the conclusions?
The manuscript must describe a technically sound piece of scientific research with data that supports the conclusions. Experiments must have been conducted rigorously, with appropriate controls, replication, and sample sizes. The conclusions must be drawn appropriately based on the data presented.
Reviewer #1: Yes
Reviewer #2: Partly

**No response required.**

2. Has the statistical analysis been performed appropriately and rigorously?
Reviewer #1: Yes
Reviewer #2: Yes

**No response required.**

3. Have the authors made all data underlying the findings in their manuscript fully available?
The [PLOS Data policy](http://www.plosone.org/static/policies.action#sharing) requires authors to make all data underlying the findings described in their manuscript fully available without restriction, with rare exception (please refer to the Data Availability Statement in the manuscript PDF file). The data should be provided as part of the manuscript or its supporting information, or deposited to a public repository. For example, in addition to summary statistics, the data points behind means, medians and variance measures should be available. If there are restrictions on publicly sharing data—e.g. participant privacy or use of data from a third party—those must be specified.
Reviewer #1: Yes
Reviewer #2: No

**Data is provided as supporting file.**

4. Is the manuscript presented in an intelligible fashion and written in standard English?

PLOS ONE does not copyedit accepted manuscripts, so the language in submitted articles must be clear, correct, and unambiguous. Any typographical or grammatical errors should be corrected at revision, so please note any specific errors here.
Reviewer #1: Yes
Reviewer #2: No

**Corrected after rigorous proof-reading.**

**Reviews**

| **Comments** | **Response** |
| --- | --- |
| **Editor’s Comments**   1. When submitting your revision, we need you   to address these additional requirements. Please ensure that your  manuscript meets PLOS ONE's style requirements,  including those for file naming. The PLOS ONE style templates  can be found at <http://www.journals.plos.org/plosone/s/file?id=wj>  Vg/PLOSOne_formatting_sample_main_body.pdf and <http://www.journals.plos.org/plosone/s/file?id=ba62/>  PLOSOne_formatting_sample_title_authors_affiliations.pdf | Edited accordingly. |
| 2. Thank you for your ethics statement: "Written informed consent was taken to record their willingness. Informed consent was provided by the departmental ethics review committee, which was developed according to ethical standards of American Psychological Association." - Please amend your current ethics statement to include the full name of the ethics committee/institutional review board(s) that approved your specific study. - Once you have amended this/these statement(s) in the Methods section of the manuscript, please add the same text to the “Ethics Statement” field of the submission form (via “Edit Submission”). For additional information about PLOS ONE ethical requirements for human subjects research, please refer to http://journals.plos.org/plosone/s/submission-guidelines#loc-human-subjects-research. | Added in ethics statement (Line 235- 236). |
| 3. We note that you have indicated that data from this study are available upon request. PLOS only allows data to be available upon request if there are legal or ethical restrictions on sharing data publicly. For more information on unacceptable data access restrictions, please see http://journals.plos.org/plosone/s/data-availability#loc-unacceptable-data-access-restrictions.  In your revised cover letter, please address the following prompts:  a) If there are ethical or legal restrictions on sharing a de-identified data set, please explain them in detail (e.g., data contain potentially sensitive information, data are owned by a third-party organization, etc.) and who has imposed them (e.g., an ethics committee). Please also provide contact information for a data access committee, ethics committee, or other institutional body to which data requests may be sent.  b) If there are no restrictions, please upload the minimal anonymized data set necessary to replicate your study findings as either Supporting Information files or to a stable, public repository and provide us with the relevant URLs, DOIs, or accession numbers. For a list of acceptable repositories, please see http://journals.plos.org/plosone/s/data-availability#loc-recommended-repositories.  We will update your Data Availability statement on your behalf to reflect the information you provide. | “b) If there are no restrictions, please upload the minimal anonymized data set necessary to replicate your study findings as either Supporting Information files or to a stable, public repository and provide us with the relevant URLs, DOIs, or accession numbers.” is the choice suitable.  Data is uploaded as supporting file. |
| Additionally, I would like to add one more comment regarding the literature review, which ignores the enormous literature on gender differences in various domains of morality. For example: gender differences in moral judgments in moral dilemmas (Fumagalli et al. 2010; Friesdorf et al. 2015; Capraro & Sippel, 2017), gender differences in honesty (Capraro, 2018; Abeler et al. 2019; Gerlach et al. 2019); gender differences in cooperation (Rand, 2017); gender differences in altruism (Rand et al. 2016; Branas-Garza et al. 2018). Of course, it is not a requirement to cite these specific works, but I think that, anyway, the literature review on gender differences should be highly improved. I am looking forward for the revision. | Added in the literature review (Line 109- 127) |
| **Comments Received on December 23^rd^, 2019**  In your ethics statement you write: "Written informed consent was taken to record their willingness. Informed consent was provided by the Departmental Ethics Review Committee, Department of Humanities, COMSATS University Islamabad, which was developed according to ethical standards of American Psychological Association." Please specify whether the Departmental Ethics Review Committee, Department of Humanities, COMSATS University Islamabad approved the current study. | Added in Line 235-236 |
| **Editor’s Comments Received on January 2^nd^, 2020**  Thank you for the additional edits. For clarity and to avoid confusion, we recommend removing the statement reading, "Informed consent was provided by the Departmental Ethics Review Committee, Department of Humanities, COMSATS University Islamabad, which was developed according to ethical standards of American Psychological Association."  Also, in the manuscript, you note a consent form is provided in Appendix A, but we cannot find a consent form or an appendix. Please update this before we proceed. | Removed as directed. Attached informed consent in the end as Appendix A. |

| **Reviewer 1** | |
| --- | --- |
| Nevertheless I was surprised by the absence of any mention to the debate about the cross-cultural differences in the people morality judgement (e.g. Graham et al., 2011 for East- West differences in foundation endorsement, even if as the effect sizes in their results showed, the gender differences were much stronger than the differences between Eastern and Western cultures).  It could be necessary for introducing this study to propose some considerations and references about the main characteristics of Pakistani culture which certainly influences the relationships between genders and may partly explain possible differences across gender stereotypes. In fact expectations about the specificity of cultural context of participants are missing, nor this relevant aspect has been sufficiently covered in the discussion. At last in the Introduction Section would be useful to refer to empathy disposition, as a recognized discriminant factor between several cross-gender dimensions. | Added in introduction (Line 84-108 & Line 155- 159).  Added in discussion (Lines 361- 364, 398- 405). |
| Moreover, I have concerns about some aspects of the methodological approach. 1. The choice of the sample, constituted in prevalence by university students, is not representative of the Pakistani population. This must be acknowledged among the limitations of the study; 2. Alpha coefficients for single scale in the sample examined are missing; | 1. Added in discussion (Lines 422- 427). 2. Added in results (Line 266- 273)   Added in discussion (Lines: 331-358). |
| Minor points: 1. Why in all tables the average scores (Actual and Stereotypes scores) of Males (MA vs, SAM) and Females (SAF vs. FA) are reported in a different order? This is confusing. 2. The labels of the 5 moral Foundations are differently used in the different parties of the text. For example the Harm/Care sub-scale in prevalence is called simply Care sub-scale, sometimes it is called Harm sub-scale. On the other side, Graham et al. in their validation study of MFQ (2011) called the 5 sub-scales using the first label for each couple (Harm, Fairness, In-group, Authority, Purity). For more clarity and consistency in the interpretation of the results obtained, according to my opinion it would be better using always the complete labels of the 5 moral bi-dimensional Foundations (Harm/Care, Fairness/Reciprocity, In-group/Loyalty, Authority/Respect, Purity/Sanctity). | 1.Corrected in all tables as “MA = Men Actual, SAM = Stereotype About Men, WA = Women Actual, SAW = Stereotype About Women.”  2. Corrected throughout text as “Harm, Fairness, In-group, Authority, Purity” according to Graham et al. (2011). |

| **Reviewer 2**  I have however some concerns, regarding, in particular, how the study is presented at the conceptual level. Indeed, I do not understand why the study is presented as a research on stereotype accuracy. According to Jussim et al. (2015), who are cited in the manuscript, the assessment of stereotype accuracy implies a comparison between people’s beliefs about a group and some criteria that establish group characteristics (i.e., more objective data such Census data about the proportion of people who have certain characteristics or perform specific behaviors). In the present manuscript, such a comparison is not done. If I understood well, the authors claim that a “standard of comparison” can be represented by ratings of women and men about themselves. However, it seems to me that these can be intended as self-construal ratings rather than criteria that allow to grasp stereotype accuracy. This is my major concern. I think a different framework for the research would increase the impact and the readability of the paper. | Added in Introduction (Line 168-192) |
| --- | --- |
| I would also stress the importance of collecting such data in the Pakistani context, and would discuss in a deeper way how these results can be specific for that context (or can be generalized to others). This argument might also be developed in the future directions section. | Pakistani culture and other aspects relevant to the context of the research have now been mentioned in the introduction and discussion section. (Lines 84-108, 155- 159, 361- 364, 398- 405) |
| With respect to the implications of the research, the authors anticipate in the introduction the importance of exploring morality and related stereotypes in the light of the #MeToo movement. I think a similar reasoning should be moved to (and expanded in) the discussion. Moreover, I find that the very last paragraph of the discussion should be expanded. The authors should provide more reasoning on the practical implications of this study. | Added in discussion (Line 398- 405) |
| Minor points: I find it very hard to follow the section results due to the numerous acronyms used in the Tables (MA, SAM, SAF, FA) I am not an English-native speaker, but I have the impression that the manuscript would benefit from a linguistic revision. Moreover, to my knowledge the APA style does not use male and female as nouns, but only as adjectives. I would rather use women and men as nouns. | 1. Corrected in all tables as “MA = Men Actual, SAM = Stereotype About Men, WA = Women Actual, SAW = Stereotype About Women.”  2. Corrected as Men & Women across paper. |
