# Supplementary material for: Accuracy of consensual stereotypes in moral foundations: A gender analysis
Source: PLoS One. 2020 Mar 5;15(3):e0229926. doi: 10.1371/journal.pone.0229926 (PMC7058411; doi:10.1371/journal.pone.0229926)
Supplement: Attachment — Submitted filename: Response to reviewers.docx [file pone.0229926.s004.docx]

**Rebuttal Letter**

**Comments**

**Reviewer #1:** (No Response)
**Reviewer #2:**

**In the revised version, the authors have addressed the points raised by me and the other reviewer. In particular, I appreciated the adding of more considerations and references to the Pakistani context, which help understand and contextualize the findings.
I only have few concerns, which are outlined below.
With respect to gender differences in morality, the authors mostly reported studies on moral judgment. Even though I agree that there has been little research on morality in gender stereotypes, I suggest that the authors look at and eventually add the evidence of Leach, Carraro, Garcia, & Kang (2017) on stereotype of women as more trustworthy than men; Sheppard and Johnson (2019) on attractiveness and trustworthiness; Moscatelli, Menegatti et al. (2020, advanced online publication on Sex Roles) on the importance of morality for women in employment evaluation. Other studies that have shown differences in morality between women and men are Franke, Crown, & Spake, 1997; Lippa, 1998; Muehlheusser, Roider, & Wallmeier, 2015.**
Added in introduction (Line 75- 98; 107-112) and References (Line 516-524; 532-536).

**Basing on the mentioned evidence, I would be more cautious than saying “…since no published work could be found on the topic of moral stereotypes and gender” (lines 152-154). I would rather say that** **the evidence on this issue is rare.**

Corrected Line 181-183. **At the end of the introduction, the authors highlighted some limitations of the study. While I appreciate this part, I think they should just mention these limitations and discuss them more in depth (for instance, the last sentence, line 189-192) in the limitations section in the discussion (which is embedded in the text at the moment).**
Line 189-192 removed from introduction. Limitations and recommendations is made as a separate section (Line 448) and is discussed in detail (Line 449 - 454).

**I would rename the section “Materials and Methods” as “Method”.**

Corrected Line 219.
**In the discussion, line 343, I cannot understand the sentence “…where people could large amounts of…”**

Corrected Line 369**.
I would avoid reporting statistics in the discussion (d values), could they be moved to the results section?**

Removed from discussion section.
